# Analysis of Antioxidant Consumption, Body Mass Index and the Waist-Hip Ratio in Early Postmenopause

**DOI:** 10.3390/medsci7010004

**Published:** 2019-01-03

**Authors:** Carlos A. Jiménez-Zamarripa, Liliana Anguiano-Robledo, Patricia Loranca-Moreno, M. Esther Ocharan-Hernández, Claudia C. Calzada-Mendoza

**Affiliations:** 1 Instituto Politécnico Nacional-ESM. Plan de San Luis y Díaz Mirón S/N, Col. Casco de Santo Tomás, Delegación Miguel Hidalgo, Mexico City C.P. 11340, Mexico; carlosajz@msn.com (C.A.J.-Z.); languianorobledo@yahoo.com.mx (L.A.-R.); paty_lm2502@hotmail.com (P.L.-M.); estherocharan@hotmail.com (M.E.O.-H.); 2 Hospital Psiquiátrico “Samuel Ramírez Moreno”-SSA. Autopista Mex-Puebla, No.83 Col. Ampliación Santa Catarina, Valle de Chalco Solidaridad, Mexico City C.P. 56619, Mexico; 3 Clínica de Peri-postmenopausia y metabolismo óseo. Hospital Regional 1° de Octubre ISSSTE. Av. Instituto Politécnico Nacional #1669 Col. Magdalena de las Salinas. Delegación Gustavo A. Madero, Mexico City C.P. 07760, Mexico

**Keywords:** postmenopause, body mass index, waist-hip ratio, antioxidants

## Abstract

Oxidative stress is present in early postmenopause. Antioxidants, present in food, avoid or limit the damage caused by free radicals. The aim of this study was to analyze whether the consumption of vitamin A, vitamin C, and Selenium was adequate in postmenopausal women and its relationship with levels of malondialdehyde. A descriptive, cross-sectional prospective clinical study was carried out with 132 women (45–55 years old) in postmenopause. The body mass index (BMI) and the waist-to-hip ratio (WHR) were calculated. The participants were surveyed about their food consumption for seven days. The plasmatic concentration of malondialdehyde was quantified by the methyl-phenyl-indole method. The women were grouped according to their BMI. All groups showed similar consumption of proteins, lipids, and carbohydrates, which exceeded the daily recommended level. According to the WHR, 87% had android fat distribution. Selenium, vitamin C, and vitamin A intake were below the daily recommended/suggested levels. The greater the BMI, the higher the plasmatic concentration of malondialdehyde in the patients. It was observed an elevated caloric intake, android fat distribution, and a greater BMI was accompanied by a lower consumption of antioxidants and an increased level of malondialdehyde.

## 1. Introduction

Early postmenopause is the period immediately after menopause and lasts for about five years [1]. A decrease in the production of estradiol in women triggers menopause and its symptoms. This hormone participates in various physiological processes, including calcium accretion in bones and maintenance of muscle mass and strength [2], as well as cognition, vascular tone and the plasmatic lipid profile [3,4]. In early postmenopause, women present characteristic signs and symptoms due to endocrine, physiological, and psychological changes [5,6]. 

Recently, oxidative stress has been recognized as clinically relevant in the etiology of diseases such osteoporosis, vascular diseases, inflammation, cognitive deterioration, ageing, and cancer, which tend to manifest themselves during menopause and postmenopause [7]. This oxidative imbalance is exacerbated by android fat distribution and other pro-oxidant factors (e.g., pollution, smoking, greater fat consumption, and reduced antioxidant intake in the diet) [8,9].

It is known that in the stage of early postmenopause, exercise and an adequate diet are essential factors for controlling hypertension, insulin resistance, and obesity [10]. However, it is important to emphasize that in clinical practice, no specific adjustments have yet been made in the consumption of antioxidants during postmenopause, even when women suffer from obesity or another comorbidity.

Oxidative stress has been related to cell damage, since free radicals react with proteins to form carbonyls, with lipids to form malondialdehyde, and with DNA to form 8-hydroxy deoxyguanosine. The irreversible changes in these biomolecules caused by free radicals can lead to tissue damage, ageing, cancer, and cell death [11].

Free radicals are controlled directly or indirectly by antioxidants, both those produced endogenously and those from exogenous sources like food or supplements. Endogenous free radicals play an important role in the defense of the organism against viral and bacterial infection. The level of these molecules is exacerbated with some diseases and conditions (e.g., strenuous exercise and menopause), as well as with environmental pollution, smoking, a high-fat diet, frequent consumption of food containing preservatives, and reduced consumption of food containing antioxidants [8,12,13]. The plasma level of malondialdehyde is regulated by different conditions such as the degree of absorption of vitamins in the intestine and therefore the integrity of this, moreover, prolonged supplementation can reduce the expression of the ascorbic acid transporter [14]. It has also been reported that the concentration of this biomarker increases in pulmonary, cardiac, renal, and rheumatoid arthritis diseases, among others [15,16,17].

## 2. Materials and Methods

An observational, descriptive, cross-sectional and prospective study was carried out with women from 45–50 years of age in postmenopause that were given medical attention in the Women’s Unit, Climacteric Clinic, Ignacio Zaragoza General Hospital of the ISSSTE (Instituto de Seguridad y Servicios Sociales de los Trabajadores del Estado). The exclusion criteria were the consumption of food supplements, acetylcysteine, green tea, black tea, and wine. On the other hand, acetaminophen, neomycin, polymyxin B, kanamycin, gentamicin, and chloramphenicol; as well as smoking and alcoholism, uncontrolled chronic degenerative disease, cancer, recent heart attack, or endocrine disorders. 

The clinical histories of the participants were recorded, the systolic, diastolic, and mean blood pressure taken. Anthropometric parameters were measured based on the International Society for the Advancement of Kinanthropometry, which included weight, height, and waist and hip circumference. Calculation was made of the BMI, and the waist-to-hip ratio (WHR) [18]. The amount of nutrients was calculated from the frequency of food consumption of seven days [19], based on the tables of composition of food and Mexican food products [20], of the National Institute of Food Medical Sciences and Nutrition, Salvador Zubirán. Finally, plasma was obtained from peripheral blood for the quantification of malondialdehyde (by the methyl-phenyl-indole method) as a marker of oxidative damage [21,22]. Briefly, the plasma was mixed with the solution of 1-methyl-2-phenylindole (10 mM), prepared in acetonitrile/methanol, which was incubated for 40 min at 45 °C, then hydrochloric acid was added, and after 5 min it was centrifuged and read at 586 nm.

The present study was authorized by the Research and Ethics Committee of the Ignacio Zaragoza hospital of the ISSSTE with code 37/2018. Informed consent was signed by all participants (after being given a thorough explanation of the protocol) before any procedures were carried out. The protocol complies with the rules of the Declaration of Helsinki of 1975, revised in 2013 in Fortaleza, Brazil. The data were processed by using descriptive and inferential statistics with parametric and non-parametric analysis (according to the specific case) on Sigma Stat version 3.2 (Systat Software, Inc., San Jose, CA, USA), considering statistical significance at *p* < 0.05.

## 3. Results

In the present study, the 132 participating women had the following parameters (average value): Blood pressure at 120/80 mmHg, age of menarche of 13 years, and menopause at the age of 46. All women had glucose and cholesterol values within the normal range. The majority of the women suffered from high blood pressure but were under medical care (Table 1).

The patients were grouped by BMI to compare this parameter to the intake of kilocalories and macronutrients. In all groups, consumption of food exceeded the recommended level, but there were no significant differences between groups (Table 2).

The comparison of the WHR of each group showed a greater percentage of women with android versus gynoid fat distribution (Table 3).

Regarding antioxidants and the concentration of malondialdehyde (a marker of damage to lipids by oxidative stress), a tendency was observed to lower antioxidant intake and a higher level of damage to lipids in participants having a greater BMI (Table 4).

Finally, the correlation test was performed between the plasmatic malondialdehyde concentration and the quantity of antioxidants consumed, finding the following values: Selenium, *r* = 2.94 × 10^−8^, *p* = 0.612; vitamin A, *r* = 0.0130, *p* = 0.300; and vitamin C, *r* = 1.40 × 10^−4^, *p* = 0.446. No correlation was found.

## 4. Discussion

The participating postmenopausal women were grouped by BMI. Analysis was made of the macronutrient and antioxidant intake, as well as the plasmatic concentration of malondialdehyde. 

The average age of menarche (13.04 ± 1.98 years) was similar to that documented in Mexican populations [23]. The average age of menopause (46.90 ± 6.81 years) is within the range reported around the world (45–55 years) and reflects the generally earlier menopause in Latin women (~47 years) compared to European women (52–55 years) [18,22,24].

Average systolic and diastolic blood pressure was 116.95 ± 11.05 and 74 ± 13.51, respectively. Considering the standard deviation, both readings are elevated according to the guide published in 2017 [25], which describes <120/80 mmHg as normal and 120–129/<80 mmHg as high. The data on the waist-to-hip ratio revealed that the majority of the patients (87%, independently of the category of BMI) had android fat distribution (Table 3), based on the standard of 0.8 as limit for gynecoid fat distribution [26,27]. This standard represents the point at which there is a risk factor for developing dyslipidemia, diabetes mellitus, and hypertension. All of the latter can trigger cardiovascular disorders [28,29]. 

The biochemical analysis of blood parameters showed an average concentration of glucose at 96.41 ± 14.66 mg/dL and cholesterol at 207.37 ± 33.03 mg/dL. These levels are within the normal range, being 70–100 mg/dL [30] for glucose and 200 mg/dL for cholesterol. The latter value may be indicative of the onset of dyslipidemia and is a risk factor for atherosclerosis. [31]).

The caloric consumption of all women (regardless of the BMI group) was greater than the recommended daily intake of 1800 kcal, which represents an excess of 308 for the eutrophic participants, 258 for those overweight, and 292 and 388 for class I and II obesity patients, respectively. Likewise, the consumption of proteins was above the recommended level of 68 g/day for all groups, elevated by 11, 9, 10, and 12 g, respectively. The intake of lipids, recommended at 60 g per day, was in excess by 36, 30, 32, and 38 g per group, respectively (Table 2), and the consumption of carbohydrates was higher than the recommended 248 g per day by 14, 12, 12, and 31 g per group, respectively. This overconsumption predisposes an individual to accumulate abdominal and visceral fat, leading to the development of obesity and vascular diseases [32].

Interestingly, the lack of differences in macronutrient intake between groups could possibly mean that the participants did not report all food eaten. Assuming all patients ate the same type of food in similar quantities, the diverse BMI levels would owe themselves to distinct hygiene/dietary habits, family genetic background, and the type, intensity, and time dedicated to exercise. Given the predisposition to a weight increase and dyslipidemia during menopause and postmenopause, a boost in the intake of soluble fiber and complex carbohydrates, and a reduction in simple carbohydrates has been recommended for women in these stages of life, while taking care to maintain an adequate balance of lipids, especially omega 3 and omega 6 fatty acids [33]. The subjects of the present study did not follow the aforementioned recommendations. 

The results demonstrate that among the current participants, the greater the BMI, the lower the antioxidant consumption. Even in the eutrophic group, the daily intake of vitamin A, vitamin C, and selenium was less than the recommended level. 

The intake of vitamin A was below the recommended daily dose (700 µg) in all groups, being 546 ± 181 µg for the eutrophic and overweight women, and 549 ± 268 and 523 ± 233 µg for class I and II obesity patients, respectively. This situation could contribute to oxidative stress, since vitamin A neutralizes singlet oxygen, captures free radicals produced in the skin due to exposure to ultraviolet radiation, and regenerates the oxidated form of vitamin C. Additionally, it can inhibit lipid peroxidation of low-density lipoprotein (LDL) cholesterol while enhancing the quantity of high-density lipoprotein (HDL) cholesterol [34].

The intake of vitamin C was only slightly less in all groups (except class I obesity) than the daily recommended dose of 75 mg, which probably owes itself to the ample access of this vitamin in fruit and especially in some that are commonly consumed, such as strawberries, guavas, and lemons/limes. The function of vitamin C is to regenerate the oxidated form of vitamin E and capture hydroxyl radicals. In addition, vitamin C acts as an antioxidant in the vascular endothelium, where it recycles cofactor BH4, which is in charge of maintaining Ferrous iron in the catalytic site of nitric oxide synthase, therefore promoting the activity of the enzyme [34,35,36].

In all groups, the intake of selenium was about half of the recommended daily amount (48 µg). The maximum level observed herein was for the eutrophic women with 24 ± 19 µg/day (Table 4). Selenium is an essential micronutrient that acts as a cofactor of antioxidant enzymes (e.g., glutathione peroxidase) and reduces oxidated vitamin E [35]. Women in postmenopause and with dyslipidemia are known to have a lower serum concentration of selenium [37]. 

As can be appreciated, the participants in the current study did not consume enough antioxidants during their postmenopause stage. Although antioxidant intake has been recommended, further research is needed to establish the adequate quantity according to the particular BMI of an individual and the type (central or visceral) of obesity. 

Upon comparing the plasmatic concentration of malondialdehyde between the distinct groups classified by BMI, no significant difference was found because of the ample variability of this marker within each group. However, there was a tendency to a higher level in the patients having a greater BMI, which has been previously reported in the Mexican population [35]. On the other hand, no significant correlation was detected between antioxidant intake and the plasmatic concentration of malondialdehyde, probably due to multiple factors, both endogenous and exogenous; that contribute to the antioxidant defense. Adequate consumption of antioxidants is necessary to reduce oxidative stress when this condition is involved in the physiopathological mechanism of disease (e.g., overweight, obesity, hypertension, insulin resistance, and dyslipidemia) [38]. These disorders are the triggers of metabolic syndrome. 

## 5. Conclusions

The diet of the population presently under study was hypercaloric. Independently of the BMI, 87% of the participants displayed an android fat distribution. Regarding the recommended daily intake of nutrients (proteins, lipids, and carbohydrates), the excess consumption in the four groups was similar. Due to the ample standard deviations among individuals with class I obesity, no significant difference existed in the plasmatic level of malondialdehyde between this group and the others. Nevertheless, there was a clear tendency to an increase for the class II obesity patients compared to the other groups. No correlation was found between the concentration of malondialdehyde and the intake of nutrients or antioxidants.

## Figures and Tables

**Table 1:** Demographic variables of the current population.

Parameter	Mean
**Age of menarche (years)**	13 ± 2
**Age of menopause (years)**	47 ± 7
**Pregnancies**	2 ± 1
**Births given**	1
**Systolic blood pressure (mmHg)**	117 ± 11
**Diastolic blood pressure (mmHg)**	74 ± 13
**Glucose (mg/dL)**	96 ± 15
**Cholesterol (mg/dL)**	207 ± 33

Data are expressed as the mean ± standard deviation.

**Table 2:** Intake of macronutrients.

Nutrient	R	Eutrophic (X ± SD)	Overweight (X ± SD)	Obesity 1 (X ± SD)	Obesity 2 (X ± SD)	*p*
Kilocalories (kcal)	1800	2108 ± 266	2058 ± 328	2092 ± 273	2188 ± 229	0.88
Proteins (g)	68	79 ± 13	77 ± 15	78 ± 14	80 ± 8	0.94
Lipids (g)	60	96 ± 13	90 ± 16	92 ± 12	98 ± 12	0.97
Carbohydrates (g)	248	262 ± 46	260 ± 54	260 ± 45	279 ± 19	0.61

*p* > 0.05, one-way ANOVA. R = recommended daily dose; obesity 1 = class I obesity; obesity 2 = class II obesity. X = Mean, SD = standard deviation.

**Table 3:** Percentage of patients with gynoid or android fat distribution, classified by body mass index (BMI).

BMI	WHR (<0.8) Gynoid	WHR (≥0.8) Android
**Normal (*n* = 27)**	7.5%	92.5%
**Overweight (*n* = 51)**	16%	84%
**Class I obesity (*n* = 29)**	7%	93%
**Class II obesity (*n* = 8)**	37.5%	62.5%

WHR = waist-to-hip ratio.

**Table 4:** Comparison of the consumption of antioxidants and the plasmatic level of malondialdehyde in each BMI category.

Antioxidants	R	Eutrophic (X ± SD)	Overweight (X ± SD)	Obesity 1 (X ± SDE)	Obesity 2 (X ± SD)	*p*
Vitamin A (µg)	700 ^1^	804 ± 618	546 ± 181	549 ± 268	523 ± 233	0.7
Vitamin C (mg)	75 ^2^	61 ± 44	67 ± 58	145 ± 122	60 ± 33	0.6
Selenium (µg)	48 ^2^	24 ± 19	21 ± 12	22 ± 13	20 ± 12	0.34
MDA (mM)	---	5.11 ± 2.29	5.66 ± 2.80	6.05 ± 5.17	11.6 ± 0.58	0.53

R = Recommended daily dose; Obesity 1 = class I obesity; Obesity 2 = class II obesity; MDA = malondialdehyde. X = mean, SD = standard deviation.
